# Supplementary material for: Deep Learning Predicts Total Knee Replacement from Magnetic Resonance Images
Source: Sci Rep. 2020 Apr 14;10:6371. doi: 10.1038/s41598-020-63395-9 (PMC7156761; doi:10.1038/s41598-020-63395-9)
Supplement: Supplementary file 1 — Supplementary Information. [file 41598_2020_63395_MOESM1_ESM.pdf]

Deep Learning Predicts Total Knee Replacement from Magnetic Resonance Images

Aniket A. Tolpadi[12], Jinhee J. Lee[2], Valentina Pedoia[2], Sharmila Majumdar[2*]
[1]Department of Bioengineering, University of California, Berkeley, USA
[2]Department of Radiology and Biomedical Imaging, University of California, San Francisco, USA
**Correspondence should be addressed to S.M. (email: Sharmila.Majumdar@ucsf.edu)*

SUPPLEMENTAL FIGURES

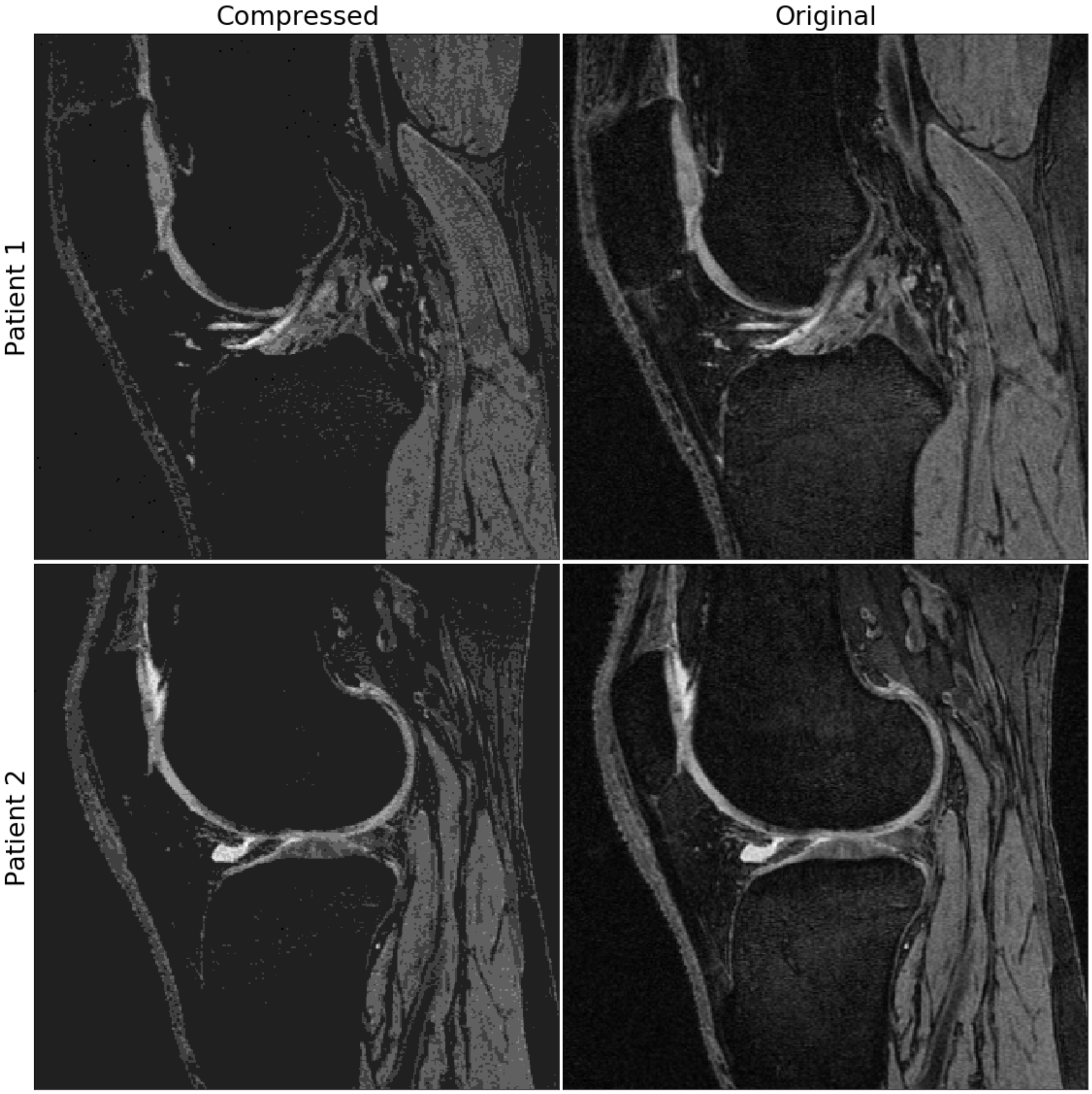

Supplementary Figure S1: Sample slices of DESS MRI and their corresponding compressed versions when rounding pixel values after normalization.

SUPPLEMENTAL TABLES

Supplementary Table S1: Non-imaging variables identified from literature as correlated with OA progression or eventual TKR. These non-imaging variables were taken to the OAI database, and, if present, added as potential non-imaging variables to supplement image-based predictions.

Variable grouping	Variable	Source
Demographics	Age	(Lewis, 2013) [22]
	Obesity/BMI	(Lewis, 2013) [22]
	Gender	(Heidari 2011) [34]
	Ethnicity	(Yu, 2019) [23]
	Income	(Hawker, 2006) [21]
	Education level	(Pisters, 2012) [36]
Previous knee trauma and pain	Knee pain	(Lewis, 2013) [22]
	Previous knee trauma	(Heidari 2011) [34]
	Repetitive knee trauma	(Heidari 2011) [34]
	Previous meniscal injuries	(Heidari 2011) [34]
	Previous knee injury	(Cooper, 2000) [35]
Knee physical activity and functionality	Mechanical forces exerted on knee	(Heidari 2011) [34]
	Frequent kneeling	(Heidari 2011) [34]
	Frequent squatting	(Heidari 2011) [34]
	Physical activity level	(Pisters, 2012) [36]
	Muscular weakness	(Heidari 2011) [34]
	Joint range of motion	(Pisters, 2012) [36]
	Lower knee extension muscle strength	(Pisters, 2012) [36]
Previous actions to treat knee pain	Previous joint injections	(Yu, 2019) [23]
	Previous knee arthroscopy	(Yu, 2019) [23]
	Previous analgesics or opioid usage	(Lewis, 2013) [22]
	Previous NSAID usage	(Yu, 2019) [23]
	Number of previous knee referrals	(Yu, 2019) [23]
	Number of previous consultations	(Yu, 2019) [23]
	Willingness to consider TJA as treatment	(Hawker, 2006) [21]
	Seen physician for arthritis in previous year	(Hawker, 2006) [21]
Preexisting health conditions	Heberden's nodes	(Cooper, 2000) [35]
	Recorded diagnosis of joint-specific OA	(Yu, 2019) [23]
	Low back pain	(Yu, 2019) [23]
	Hypertension	(Yu, 2019) [23]
	Smoking status	(Yu, 2019) [23]
	Drinking status	(Yu, 2019) [23]
	Asthma	(Yu, 2019) [23]
	COPD	(Yu, 2019) [23]
	Diabetes mellitus	(Yu, 2019) [23]
	Comorbidities	(Pisters, 2012) [36]
Miscellaneous	Knee joint laxity	(Heidari 2011) [34]
	Genetic susceptibility to knee OA	(Heidari 2011) [34]
	Mental health measures	(Sharma, 2003) [37]
	SF36 score	(Hawker, 2006) [21]

Supplementary Table S2: Percentages of selected tissues identified as hotspots among 124 true positives detected by integrated MRI pipeline, stratified by OA severity.

Tissue type	Tissue	No OA (n = 11)	Moderate OA (n = 65)	Severe OA (n = 48)	Total (n = 124)
Cartilage	TFJ medial	100.0	95.4	87.5	92.7
	TFJ lateral	100.0	87.7	85.4	87.9
	PFJ	27.3	43.1	41.7	41.1
Meniscus	Medial anterior	100.0	84.6	75.0	82.3
	Medial posterior	90.9	87.7	70.8	81.5
	Lateral anterior	100.0	87.7	81.3	86.3
	Lateral posterior	100.0	90.8	81.3	87.9
Bone	TFJ medial	100.0	95.4	89.6	93.5
	TFJ lateral	90.9	87.7	83.3	86.3
	PFJ	27.3	35.4	45.8	38.7
Ligament	ACL	100.0	81.5	64.6	76.6
	PCL	72.7	73.8	77.1	75.0
	Popliteal	54.5	56.9	58.3	57.3
Tendon	Medial patellar retinaculum	90.9	78.5	91.7	84.7
	Lateral patellar retinaculum	54.5	21.5	33.3	29.0
	Popliteal	36.4	49.2	43.8	46.0
	Patellar	27.3	27.7	25.0	26.6
	Gastrocnemius	36.4	9.2	14.6	13.7
	Semimembranosus	27.3	13.8	6.3	12.1
	Quadriceps	0.0	4.6	14.6	8.1
	Gracilis	0.0	4.6	6.3	4.8
Fat pad	Hoffa	100.0	90.8	97.9	94.4
Muscle	Popliteus	18.2	35.4	10.4	24.2
	Vastus medialis	18.2	7.7	18.8	12.9
	Gastrocnemius	36.4	26.2	27.1	27.4
	Plantaris	27.3	32.3	31.3	31.5
	Biceps femoris	0.0	4.6	6.3	4.8
	Tibialis anterior	0.0	4.6	0.0	2.4
	Semimembranosus	0.0	3.1	2.1	2.4
Synovium	General	81.8	87.7	93.8	89.5

Supplementary Table S3: Percentages of selected tissues identified as hotspots among 124 true negative controls detected by integrated MRI pipeline, stratified by OA severity.

Tissue type	Tissue	No OA (n = 11)	Moderate OA (n = 65)	Severe OA (n = 48)	Total (n = 124)
Cartilage	TFJ medial	100.0	100.0	100.0	100.0
	TFJ lateral	100.0	100.0	100.0	100.0
	PFJ	18.2	27.7	62.5	40.3
Meniscus	Medial anterior	100.0	92.3	93.8	93.5
	Medial posterior	100.0	96.9	83.3	91.9
	Lateral anterior	90.9	96.9	97.9	96.8
	Lateral posterior	100.0	100.0	91.7	96.8
Bone	TFJ medial	100.0	100.0	97.9	99.2
	TFJ lateral	100.0	100.0	100.0	100.0
	PFJ	9.1	26.2	56.3	36.3
Ligament	ACL	100.0	90.8	79.2	87.1
	PCL	45.5	63.1	72.9	65.3
	Popliteal	54.5	46.2	41.7	45.2
Tendon	Medial patellar retinaculum	54.5	66.2	87.5	73.4
	Lateral patellar retinaculum	18.2	23.1	35.4	27.4
	Popliteal	45.5	33.8	37.5	36.3
	Patellar	9.1	16.9	18.8	16.9
	Gastrocnemius	0.0	9.2	0.0	4.8
	Semimembranosus	36.4	26.2	12.5	21.8
	Quadriceps	0.0	0.0	6.3	2.4
	Gracilis	0.0	1.5	0.0	0.8
Fat pad	Hoffa	81.8	81.5	95.8	87.1
Muscle	Popliteus	18.2	15.4	10.4	13.7
	Vastus medialis	0.0	7.7	16.7	10.5
	Gastrocnemius	45.5	29.2	6.3	21.8
	Plantaris	18.2	15.4	10.4	13.7
	Biceps femoris	0.0	1.5	0.0	0.8
	Tibialis anterior	9.1	0.0	0.0	0.8
	Semimembranosus	27.3	7.7	2.1	7.3
Synovium	General	90.9	87.7	87.5	87.9
